# Deletion of the N-terminus of SF2/ASF Permits RS-Domain-Independent Pre-mRNA Splicing

**DOI:** 10.1371/journal.pone.0000854

**Published:** 2007-09-05

**Authors:** Stephanie D. Shaw, Sutapa Chakrabarti, Gourisankar Ghosh, Adrian R. Krainer

**Affiliations:** 1 Cold Spring Harbor Laboratory, Cold Spring Harbor, New York, United States of America; 2 Department of Chemistry and Biochemistry, University of California at San Diego, La Jolla, California, United States of America; 3 Molecular and Cellular Biology Program, State University of New York at Stony Brook, Stony Brook, New York, United States of America; Centre de Regulació Genòmica, Spain

## Abstract

Serine/arginine-rich (SR) proteins are essential splicing factors with one or two RNA-recognition motifs (RRMs) and a C-terminal arginine- and serine-rich (RS) domain. SR proteins bind to exonic splicing enhancers via their RRM(s), and from this position are thought to promote splicing by antagonizing splicing silencers, recruiting other components of the splicing machinery through RS-RS domain interactions, and/or promoting RNA base-pairing through their RS domains. An RS domain tethered at an exonic splicing enhancer can function as a splicing activator, and RS domains play prominent roles in current models of SR protein functions. However, we previously reported that the RS domain of the SR protein SF2/ASF is dispensable for *in vitro* splicing of some pre-mRNAs. We have now extended these findings via the identification of a short inhibitory domain at the SF2/ASF N-terminus; deletion of this segment permits splicing in the absence of this SR protein's RS domain of an IgM pre-mRNA substrate previously classified as RS-domain-dependent. Deletion of the N-terminal inhibitory domain increases the splicing activity of SF2/ASF lacking its RS domain, and enhances its ability to bind pre-mRNA. Splicing of the IgM pre-mRNA in S100 complementation with SF2/ASF lacking its RS domain still requires an exonic splicing enhancer, suggesting that an SR protein RS domain is not always required for ESE-dependent splicing activation. Our data provide additional evidence that the SF2/ASF RS domain is not strictly required for constitutive splicing *in vitro*, contrary to prevailing models for how the domains of SR proteins function to promote splicing.

## Introduction

The SR proteins are a family of conserved splicing factors that consist of either one or two N-terminal RNA recognition motifs (RRM) and a C-terminal arginine- and serine-rich (RS) domain [1], [2]. SR proteins promote constitutive and alternative splicing through multiple modes [3], some of which are presumed to require their RS domains. Exonic splicing enhancers (ESEs) are degenerate 6–8 nucleotide motifs that promote exon inclusion, in many cases through the action of SR proteins [4]–[9]. SR proteins bind to ESEs via their RRM(s) [10], whereas their RS domains are thought to function as protein-protein interaction modules that facilitate exon inclusion by recruiting components of the basal splicing machinery to the flanking 5′ and 3′ splice sites early in splice-site recognition [11]. In yeast two-hybrid and Far Western assays, the SR protein SF2/ASF was shown to interact with itself and with the U1-snRNP-specific protein U1-70K and the small subunit of the U2AF heterodimer, U2AF^35^; these protein-protein interactions required the RS domains of each protein [12], [13]. Subsequently it was proposed that SR proteins can promote recruitment of the U1 snRNP to the 5′ splice site through SR protein RS-domain-mediated interactions with U1-70K [14]. However, the RS domain of SF2/ASF alone is unable to interact with U1-70K *in vitro* [15]. Enhancer-bound SR proteins are also thought to escort U2AF^65^ to the 3′ splice site polypyrimidine tract through RS-domain-mediated recruitment of U2AF^35^ [16], [17], [18]. A role for SR proteins in bringing U2AF^65^ to the polypyrimidine tract is supported by several experiments in which improving this tract can relieve the requirement for an ESE for pre-mRNAs with enhancer-dependent introns [19], [20], [21]. However, other experiments failed to detect changes in U2AF recruitment in the presence versus in the absence of an ESE [22], [23], calling into question the hypothesis that an ESE-bound RS domain is required for recruitment of U2AF^65^. Although the aforementioned functions of SR proteins are assumed to occur via RS-domain-mediated protein-protein interactions, it has not yet been demonstrated that such interactions occur in the context of a functional spliceosome [24].

A second mode by which SR proteins promote exon inclusion is by antagonizing the negative regulation conferred by exonic splicing silencers (ESSs), pre-mRNA regulatory elements that inhibit exon inclusion in both constitutive and alternative splicing [25]. Although the mechanisms by which SR proteins counteract the effects of splicing silencers are not well understood [4], their RS domains are not always required for this function, as SF2/ASF lacking its RS domain can act from the position of an HIV *tat* exon 3 ESE to antagonize an ESS present in the same exon [26].

A third mechanism by which SR proteins have been reported to promote splicing is by engaging in transient RS domain-pre-mRNA contacts during the course of splicing. An ESE-bound RS domain can interact directly with the branchpoint of an IgM substrate in the pre-spliceosomal A complex [27]. The RS domains of SR proteins bound to ESEs can also act as protein-RNA interaction modules to promote base-pairing of pre-mRNA and U5 and U6 snRNAs during the course of pre-mRNA splicing [28]. However, although it is clear that an RS domain recruited to the ESE position can function as a splicing activator, an RS domain tethered to the position of the ESE is not always required, as splicing of the ESE-dependent substrate dsx lacking its ESE can also be accomplished simply by the addition of an excess of free RS domain to nuclear extract [27], consistent with the hypothesis that the function of an SR protein may be merely to recruit any RS domain to the vicinity of the splicing signal. On the other hand, adding an RS domain peptide to nuclear extract is insufficient to promote exon inclusion for *BRCA1* pre-mRNA exon 18 lacking a functional ESE, whereas recruitment of a synthetic RS domain to the mutated ESE rescues inclusion of this exon [29].

The RS domains of SR proteins are conserved, and the serine residues within these domains are targets of phosphorylation by multiple kinases, including SRPK1 [30] and SRPK2 [31], Clk/Sty [32], and DNA topoisomerase I [33]. Phosphorylation of RS domains influences the subcellular localization of SR proteins [32], [34], [35], [36]. The phosphorylation state of the RS domain has a significant influence on SR protein function, as both hyper- and hypophosphorylated SR proteins are unable to support splicing [37], [38], [39]. SR protein RS domains were at one time thought to be indispensable for constitutive splicing *in vitro,* yet dispensable for concentration-dependent effects on alternative splice-site selection [40], [41]. However, we subsequently found that the RS domain of SF2/ASF is not required for constitutive splicing of several pre-mRNAs *in vitro*, including tat23, an ESE-dependent pre-mRNA known to be regulated by an ESS [42]. Thus, pre-mRNAs could be classified as either RS-domain-dependent or RS-domain-independent, based on their ability to be spliced with an SR protein lacking its RS domain (“ΔRS”). RS-domain-dependence was found to be related to the strength of the 3′ splice site and the requirement for U2AF^35^ [42]. IgM M1-M2 was identified as an RS-domain-dependent pre-mRNA, congruent with at least some previous reports that it is U2AF^35^-dependent and possesses relatively weak polypyrimidine-tract and branchpoint sequences [43], [44].

IgM M1-M2 has been used by several laboratories as a model substrate to explore the role(s) of ESEs in promoting pre-mRNA splicing. However, the functions of the ESE-bound SR protein in the context of the RS-domain-dependent IgM M1-M2 pre-mRNA have been controversial, and there are several competing models for the mechanism by which SF2/ASF promotes splicing at the ESE position in this substrate. In the recruitment model, SF2/ASF binds via its RS domain to U2AF^35^ to indirectly recruit U2AF^65^ to the polypyrimidine tract [21], whereas in the antagonism model, the sole function of enhancer-bound SF2/ASF is to prevent PTB from binding to a downstream ESS [45]. Some experiments with the IgM M1-M2 substrate strongly support the model for SR protein function in which an ESE-bound RS domain recruits U2AF^35^ and U2AF^65^ to the polypyrimidine tract [21]. However, other experiments detected no difference in U2AF^35^ occupancy on IgM M1-M2 in the presence and absence of the ESE [23].

The discovery that many but not all substrates could be spliced with SF2/ASF lacking its RS domain [42] suggested that SR protein functions might be subdivided into RS-domain-dependent and RS-domain-independent categories. We prepared various fragments of SF2/ASF for structural and functional studies, including versions lacking the C-terminal RS domain and/or an N-terminal extension that precedes RRM1. N-terminal and C-terminal extensions of RRMs have been demonstrated to regulate nucleic acid binding in other splicing factors [46], and we noted that SF2/ASF and some other SR proteins have N-terminal RRM extensions. We had previously characterized an N-terminally His-tagged SF2/ASF lacking the RS domain as unable to complement S100 for constitutive splicing [40], but omitting this N-terminal tag allowed the same protein to support splicing of some pre-mRNAs [42]. These precedents suggested that the natural N-terminus of SF2/ASF may influence its activity, and we therefore investigated whether the N-terminal extension preceding RRM1 had any influence on the splicing activity of ΔRS with the RS-domain-dependent substrate IgM M1-M2. Deletion of the N-terminus from ΔRS revealed that the RS domain is not required for splicing of IgM M1-M2, lending further support to our previous finding that the RS domain of SF2/ASF is sometimes dispensable for splicing *in vitro*, and calling for a reevaluation of traditional models of SR protein function.

## Results

### The RS domain of SF2/ASF is not required for splicing of IgM M1-M2 *in vitro*

**Figure 1:**
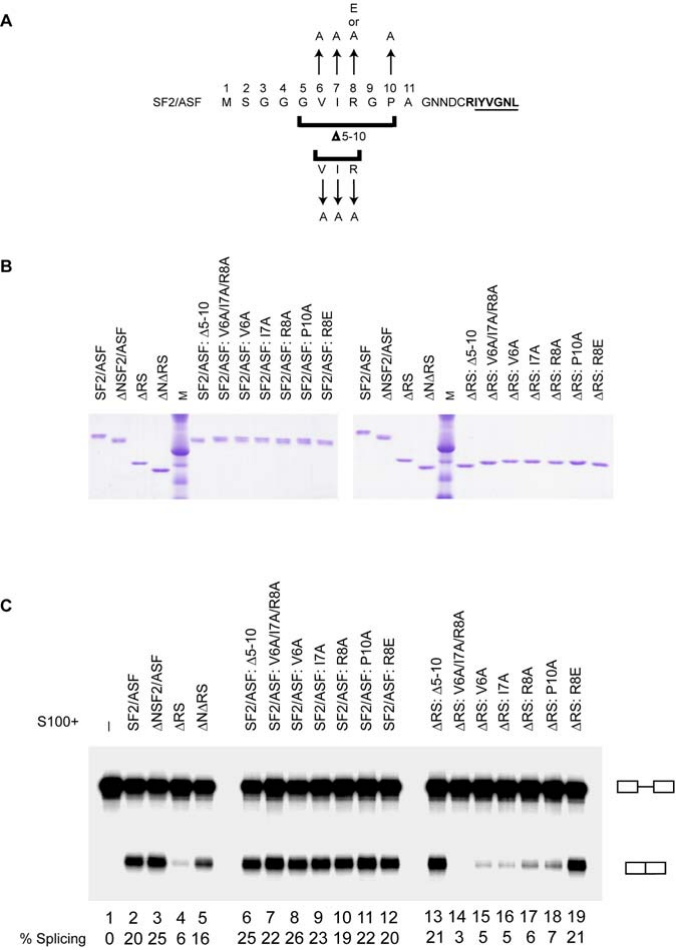
Identification of N-terminal residues of SF2/ASF that contribute to the inhibitory function of this domain. (A) Amino acid sequence of the N-terminal extension of RRM1 of SF2/ASF, indicating mutations generated and tested by *in vitro* splicing and UV crosslinking assays. The first residues of RRM1 are in bold, with the RNP-2 submotif underlined. (B) Recombinant SF2/ASF and mutant proteins used in this study, analyzed by SDS-PAGE and Coomassie-blue staining. M: molecular-weight markers. (C) *In vitro* splicing of IgM M1-M2 pre-mRNA in HeLa S100 extract alone (lane 1), and in S100 complemented with 16 pmol of SF2/ASF, ΔRS, and N-terminus mutant proteins, as indicated (lanes 2-19). The splicing efficiency is indicated below each lane.

To determine whether the splicing activity of the ΔRS protein is affected by the N-terminal extension to RRM1, we tested proteins with mutations and deletions of the N-terminus in an *in vitro* splicing assay (Figure 1). IgM M1-M2 was previously characterized as an RS-domain-dependent substrate, although a low level of splicing can be detected in S100 complementation assays with our ΔRS protein (Figure 1C, lane 4), which consists of amino acids 1-196 of SF2/ASF. Deletion of the first 11 amino acids of ΔRS to produce the ΔNΔRS protein comprising amino acids 12-196 permitted splicing of IgM M1-M2 at a level comparable to that seen with full-length SF2/ASF (Figure 1C, lanes 2 and 5). Deletion of the N-terminus from SF2/ASF to produce the ΔNSF2/ASF protein also slightly increased the amount of splicing supported by the protein (Figure 1C, lanes 2 and 3).

### Mutational analysis of the N-terminus of SF2/ASF reveals that conserved amino acids contribute to the inhibitory effect of the RRM1 extension on splicing

We observed that deletion of the 11 N-terminal amino acids (MSGGGVIRGPA) from SF2/ASF and ΔRS increased the amount of splicing that could be supported by these proteins, suggesting that the N-terminus has an inhibitory function. To identify amino acids within this N-terminal region that may contribute to inhibition of splicing, we generated SF2/ASF and ΔRS proteins with mutations in the N-terminus (Figure 1). Amino acids 5-10 (GVIRGP) are predicted to have β-strand propensity (GOR4, Biology Workbench, San Diego Supercomputer Center, University of California at San Diego; Subramaniam, 1998), and several other proteins identified through a Basic Local Alignment Search Tool search (BLAST, National Center for Biotechnology Information) as having similar motifs to GVIRGP are known to adopt a β-strand conformation with their homologous residues. We made both SF2/ASF and ΔRS proteins with the following mutations at the N-terminus: deletion of amino acids 5–10 (the predicted β-strand), a triple mutant of amino acids 6–8 called V6A/I7A/R8A, and single mutants designated V6A, I7A, R8A, P10A, and R8E (Figures 1A and 1B). The SF2/ASF and ΔRS N-terminus mutant proteins were tested in the *in vitro* splicing assay with IgM M1-M2 (Figure 1C) to determine whether mutation of any of these amino acids relieves the inhibitory effect of the N-terminus. Most of the N-terminal mutations had little or no effect on the amount of splicing of IgM M1-M2 in the context of full-length SF2/ASF (Figure 1C, lanes 6-12). However, of the ΔRS N-terminus mutant proteins, ΔRS: Δ5-10 and ΔRS:R8E showed a significant increase in splicing, relative to their parental protein ΔRS (Figure 1C, lanes 13 and 19), with levels of splicing similar to that seen with ΔNΔRS. The increase in splicing of IgM M1-M2 with these mutant proteins, relative to ΔRS, suggests that residues within amino acids 5-10 contribute to the inhibitory effect of the N-terminus on splicing, and in particular, that R8 plays a role in this inhibition.

**Figure 2:**
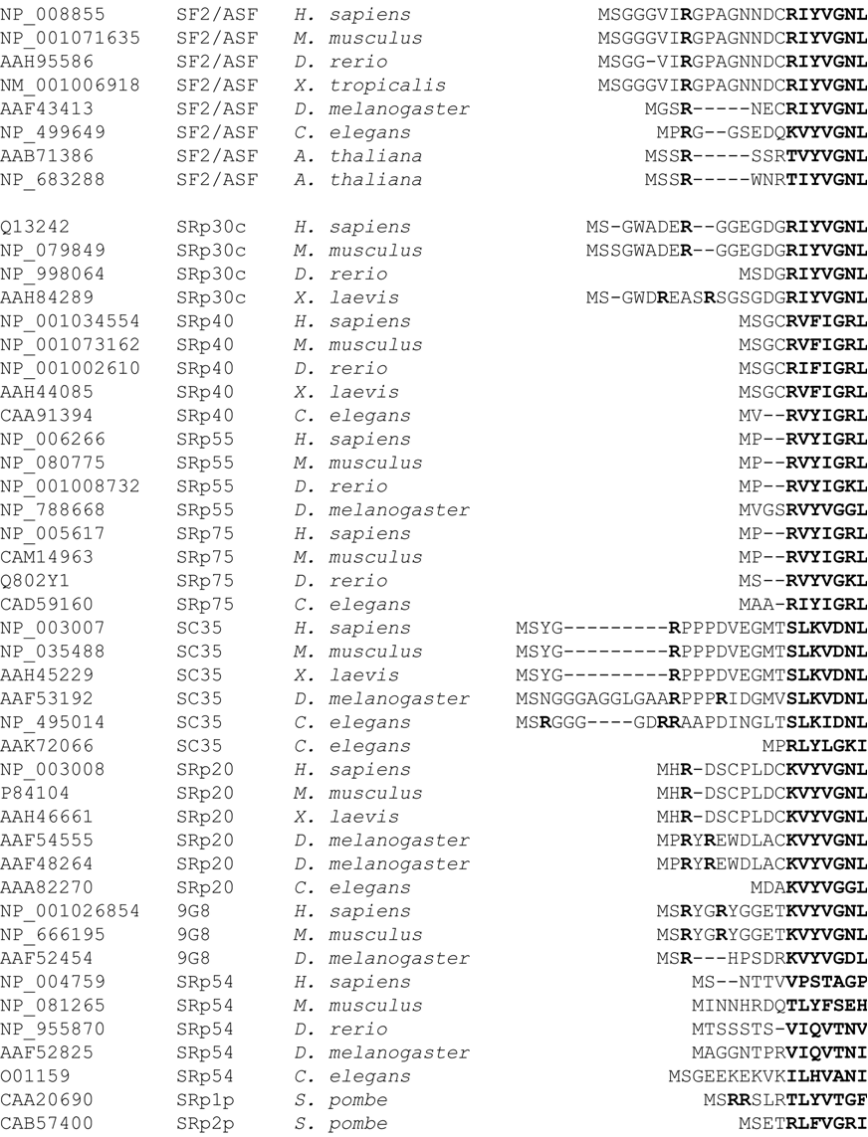
Phylogenetic alignment of the N-termini of SF2/ASF orthologs and paralogs. SR protein N-terminal RRM extensions were aligned using ClustalW. Accession numbers are provided for each sequence in the alignment. Sequences in the β1 strand and arginine residues in the extensions are indicated by bold lettering.

We carried out a phylogenetic analysis to examine the conservation of the N-terminal extension of SF2/ASF (Figure 2). The N-terminal peptide is highly conserved in vertebrate SF2/ASF orthologs, but not in other SR protein paralogs. Invertebrate and plant SF2/ASF and a subset of other SR proteins also have N-terminal extensions, which in most cases include at least one arginine residue.

### The N-terminal extension of RRM1 influences the ability of SF2/ASF to bind RNA

**Figure 3:**
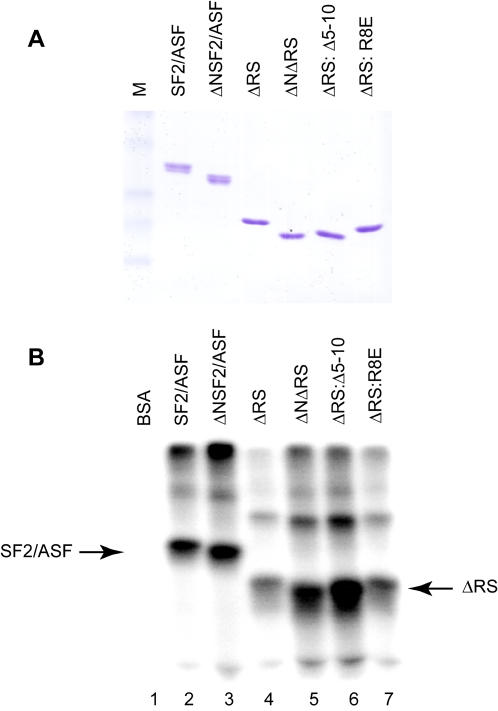
ΔRS N-terminus mutations that improve splicing also increase the ability of ΔRS to bind IgM M1-M2. (A) Recombinant SF2/ASF and mutant proteins employed in the crosslinking assay. M: molecular-weight markers. (B) UV crosslinking of SF2/ASF and variant proteins to radiolabeled IgM M1-M2 RNA. BSA (lane 1), or purified recombinant SR proteins SF2/ASF (lane 2), ΔNSF2/ASF (lane 3), ΔRS (lane 4), ΔNΔRS (lane 5), ΔRS: Δ5-10 (lane 6), and ΔRS:R8E (lane 7) were incubated with uncapped IgM M1-M2 RNA prior to crosslinking, RNAse digestion, and separation of crosslinked adducts by SDS-PAGE.

Protein segments N-terminal or C-terminal to the core RRM module have been demonstrated to play roles in nucleic acid recognition for several splicing factors, including U1-70K [47], U1A [48], [49], [50], PTB [51], hnRNP C [52], and hnRNP A1 [53], as well as other RNA-binding proteins with RRMs, such as La [54] and CstF-64 [55]. As RRM extensions modulate the specificity of RNA binding, the binding affinity, and/or the accessibility of the RNA-binding surface for other nucleic acid-binding proteins, we hypothesized that the SF2/ASF N-terminal extension may influence the affinity of the protein for splicing substrates. The binding of purified recombinant SF2/ASF, ΔNSF2/ASF, ΔRS, ΔNΔRS, ΔRS: Δ5-10, and ΔRS:R8E to IgM M1-M2 pre-mRNA was assayed by UV crosslinking (Figure 3). Although there was little difference between the extent of RNA crosslinking observed for the SF2/ASF and ΔNSF2/ASF proteins (Figure 3B, lanes 2 and 3), deletion of the N-terminal extension in the context of the ΔRS protein greatly increased the crosslinking to the IgM M1-M2 RNA (lanes 4 and 5). In addition, the ΔRS: Δ5-10 protein, which exhibited increased splicing activity relative to its parental ΔRS protein (Figure 1C, lane 13), was also more efficiently crosslinked to IgM M1-M2 RNA (Figure 3B, lane 6). These data suggest that the inhibitory effect of the N-terminus of the ΔRS protein on splicing of IgM M1-M2 may be due to a negative influence of this segment on the protein's ability to bind RNA.

The increase in splicing observed with ΔRS harboring the R8E mutation (Figure 1C, lane 19) was not strictly correlated with improvement in RNA binding as measured by UV crosslinking with purified recombinant proteins, as apparent binding of ΔRS: R8E protein to IgM M1-M2 RNA was not greatly increased relative to binding of ΔRS (Figure 3B, lanes 4 and 7). Increased apparent RNA binding in the UV crosslinking assay for ΔNΔRS and ΔRS: Δ5-10 proteins may be a consequence of removal of a portion of the inhibitory N-terminus, whereas the R8E mutation may not increase RNA binding to the same extent in this assay because it preserves the length of the inhibitory N-terminal extension to RRM1. On the other hand, the UV crosslinking assay with RNA and purified recombinant proteins does not necessarily address whether the R8E mutation affects the ability of the ΔRS protein to be recruited to IgM M1-M2 pre-mRNA under splicing conditions.

### Splicing of IgM M1-M2 with ΔNΔRS requires the exonic splicing enhancer

**Figure 4:**
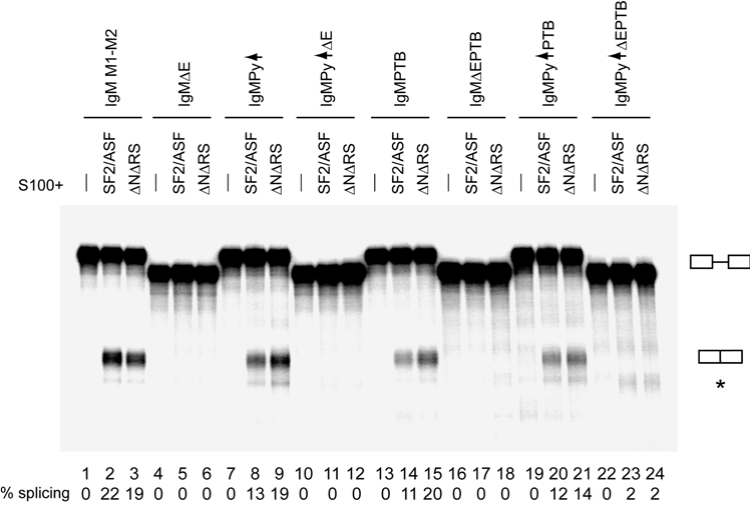
An exonic splicing enhancer is required for splicing of IgM M1-M2 with ΔNΔRS. *In vitro* splicing of IgM M1-M2 and derivative pre-mRNAs with mutations in the polypyrimidine tract, exonic splicing enhancer, and/or exonic splicing silencer: IgM M1-M2 (lanes 1-3), IgMΔE (lanes 4–6), IgMPy↑ (lanes 7–9), IgMPy↑ΔE (lanes 10–12), IgMPTB (lanes 13–15), IgMΔEPTB (lanes 16–18), IgMPy↑PTB (lanes 19–21), and IgMPy↑ΔE PTB (lanes 22–24, mRNA position indicated by asterisk); in S100 alone (lanes 1, 4, 7, 10, 13, 16, 19, and 22), and S100 complemented with 16 pmol of SF2/ASF (lanes 2, 5, 8, 11, 14, 17, 20, and 23), or ΔNΔRS (lanes 3, 6, 9, 12, 15, 18, 21, and 24). The splicing efficiency is indicated below each lane.

Despite the fact that IgM M1-M2 was previously characterized as an RS-domain-dependent substrate, and although its splicing can be activated by an RS domain tethered to the position of its ESE [21], [56], [57], we have established through deletion of the N-terminus from ΔRS that the RS domain of SF2/ASF is not required for IgM M1-M2 splicing. We and others have observed that IgM M1-M2 is an ESE-dependent substrate [7], [23], [58], [59], [60]. As several existing models for SR protein function in the IgM M1-M2 context require an enhancer-bound SR protein or RS domain, we wished to determine whether the ΔNΔRS protein exerted its effects through the ESE. Therefore, we deleted the enhancer region (GAAGGACAGCAGAGACCAAGA, as reported in [23]) from IgM M1-M2 to produce the IgMΔE substrate, and tested its ability to be spliced with the ΔNΔRS protein. We also mutated other sequences already demonstrated to have a relationship to SR protein function within the IgM M1-M2 pre-mRNA context, i.e., the polypyrimidine tract and the exonic splicing silencer, to test whether there was any difference in splicing with changes in these elements, in the presence or absence of the SF2/ASF RS domain. As already seen in the above experiment (Figure 1C), ΔNΔRS complemented S100 for splicing of IgM M1-M2 almost as efficiently as SF2/ASF (Figure 4, lanes 2 and 3). As expected, IgMΔE could not be spliced in S100 complementation with SF2/ASF, because IgM M1-M2 is an ESE-dependent pre-mRNA (Figure 4, lane 5). Likewise, ΔNΔRS could not splice IgMΔE, demonstrating that it also activates splicing in an ESE-dependent manner (Figure 4, lane 6).

The IgM M1-M2 ESS was originally identified in functional assays by progressive deletion of exonic sequences from the 3′ end of the pre-mRNA, and mapped to the last half of the M2 exon [58]. The silencer was subsequently more precisely mapped to an 11-nucleotide motif denoted PTB site I (UCUUACGUCUU), and its cognate repressor protein was identified as pyrimidine tract binding protein (PTB) [45]. Using an IgM M1-M2 derivative substrate in which the ESE had been replaced by an MS2 bacteriophage coat protein binding site [61], Shen et al showed in S100 complementation assays with SF2/ASF that immunodepletion of PTB permitted splicing of IgM in the absence of an MS2-RS protein targeted to the ESE position [45], suggesting that the primary function of an SR protein bound at this ESE is to counteract the juxtaposed ESS. To determine whether the RS domain of an ESE-bound SR protein plays a role in antagonizing the function of the IgM M1-M2 ESS, we tested an IgM substrate with a mutant PTB site I (ACAUACGACAU, as in [45]), IgMPTB, and the PTB mutant substrate also lacking the ESE, IgMΔEPTB, in S100 complementation with SF2/ASF and ΔNΔRS. More efficient splicing of IgMPTB was observed with ΔNΔRS than with SF2/ASF (Figure 4, lanes 14 and 15), suggesting that the RS domain of SF2/ASF is not required for counteracting the function of the PTB site I silencing element. However, contrary to the previous report that the PTB site I mutation can relieve the requirement for the ESE [45], we did not observe splicing of IgMΔEPTB with either SF2/ASF or ΔNΔRS in our S100 complementation assays (Figure 4, lanes 17 and 18). The discrepancies between our results and the previously reported data may be attributable to differences in the methods by which we tested the substrates; in their S100 complementation assays, Shen et al immunodepleted PTB from S100, rather than mutating the PTB site I. It is possible that mutation of the PTB site I might be insufficient to permit splicing in S100 complementation, for example, in a scenario in which PTB binds to additional sites in IgM M1-M2 to silence splicing. On the other hand, we note that our results are in agreement with other reports that an ESE is required for splicing of IgM, even in the absence of the ESS [21], [58].

We have demonstrated that ΔNΔRS, like SF2/ASF, activates splicing in an ESE-dependent manner. As improvement of the polypyrimidine tract relieves the requirement for an enhancer when the ESS is not present [21], we attempted to relieve the requirement for the ESE for splicing of IgM M1-M2 by ΔNΔRS by simultaneously improving the polypyrimidine tract and mutating the ESS. We generated the IgMPy↑ substrate, in which the polypyrimidine tract was replaced with a consensus U2AF^65^ binding site (UUUUUUCCCUUUUUUUUUC, as in [21]), and the IgMPy↑ΔE substrate, in which the enhancer was also deleted. To these substrates we added the PTB site I mutation, to create IgMPy↑PTB and IgMPy↑ΔEPTB. IgMPy↑ and IgMPy↑PTB could both be spliced in S100 complementation with either SF2/ASF or ΔNΔRS (Figure 4, lanes 8 and 9, and lanes 20 and 21), but IgMPy↑ΔE, lacking an enhancer, could not be spliced with either protein (lanes 11 and 12). Although trace amounts of splicing were detectable for IgMPy↑ΔEPTB in S100 complementation with either SF2/ASF or ΔNΔRS (Figure 4, lanes 23 and 24), we were largely unable to relieve the requirement for an ESE by simultaneously mutating the ESS and improving the polypyrimidine tract. However, there are notable differences between our experimental conditions and the manner in which the analogous experiment was carried out by Graveley et al, most important of which is that our experiment was an S100 complementation assay, rather than splicing in nuclear extract. In addition, our assay utilized IgM M1-M2 with a mutation in the PTB site I [45], rather than the IgM MS2 substrate [21], which does not have the silencer because it lacks most of exon M2 downstream of the enhancer.

We conclude from these experiments that the ESE is required for splicing of IgM M1-M2 in S100 complementation with either SF2/ASF or ΔNΔRS, suggesting that ΔNΔRS, like SF2/ASF, exerts at least some of its effects to activate splicing from the position of the exonic splicing enhancer. Significantly, our data also suggest that the RS domain is not required for enhancer-dependent SR protein functions, at least in the context of IgM M1-M2, as the levels of splicing of IgM M1-M2 and IgM-derivative substrates with ΔNΔRS were comparable to, or greater than, those observed with SF2/ASF. An RS domain tethered to the ESE position was found to be insufficient to activate splicing of an IgM substrate in S100 complementation assays, and the addition of an intact SR protein to the reaction is required for splicing to occur under these conditions [27], [45], [59]. An N-terminally His-tagged ΔRS protein was found to be unable to perform ESE-independent SR protein function(s) in this context [27]; however, we demonstrate using ΔNΔRS as the sole source of SR protein in our complementation assays that the RS domain is also not required for this additional SR protein function(s) to support splicing of IgM M1-M2.

## Discussion

### Possible mechanisms of inhibition of splicing by the N-terminus of SF2/ASF

Historically, SR protein RS domains were thought to be essential for constitutive splicing, because a recombinant SR protein lacking its RS domain was unable to complement S100 for splicing of constitutive substrates. In retrospect, what these early experiments may have been demonstrating was the importance of sequences preceding RRM1 in influencing the ability of SR proteins to be recruited to pre-mRNA. Initial experiments to test whether the RS domain of SF2/ASF is required for splicing were carried out with versions of ΔRS tagged at the N-terminus, and these experiments consistently showed that the RS domain of SF2/ASF was required for constitutive splicing [40], [41], [62]. Subsequent experiments using a version of ΔRS with no tag at the N-terminus unexpectedly showed that the RS domain was dispensable for splicing of some substrates [42]. We have not ruled out the possibility that deletion of sequences from the N-terminus, including the N-terminal extension to RRM1, increase the ability of ΔRS to bind to pre-mRNA and promote splicing merely by increasing solubility of the SR protein. Nonetheless, we now find that deletion of the majority of the amino acids preceding RRM1 in the context of the ΔRS protein permits splicing even of the IgM M1-M2 pre-mRNA, a substrate previously classified as RS-domain-dependent, further demonstrating that the RS domain is dispensable for constitutive splicing *in vitro*.

RRMs can have N-terminal and C-terminal extensions augmenting their core structure that are usually poorly ordered, but in some cases adopt a secondary structure [46]. In cases in which their functions have been documented, these N-terminal and C-terminal extensions of RRMs modulate nucleic acid binding by the core RRM. For some of these RRMs, the extensions can fold over onto the RNA-binding surface of the core domain to mask key residues involved in nucleic acid binding [54], [55], [63]. Several SR proteins, including SF2/ASF, have N-terminal extensions preceding their first RRM (Figure 2). The 20 available NMR structures of RRM1 of SF2/ASF [64] when viewed collectively hint that the N-terminal extension is probably flexible and can adopt many different conformations, such that this region could sometimes occlude key RNA-binding residues on the protein's β-sheet. The observed flexibility of the N-terminal extension could be due in part to an additional hydrophilic-tag extension that was added for the purpose of improving solubility in the structure determination.

Based on our data and the documented functions of other RRM extensions, we propose that the most likely explanation for the inhibitory effect of amino acids 2-11 of SF2/ASF on splicing is that the N-terminal segment negatively affects RNA binding by RRM1. In this model, the N-terminal domain may be inhibitory by functioning as a damper upon RRM1 to interfere with its binding to the RNA. Indeed, we have observed in UV crosslinking assays that deletion of the N-terminal extension from ΔRS greatly enhances apparent binding of the protein to IgM M1-M2 RNA (Figure 3B). These quantitative differences in crosslinking probably reflect differences in binding; although residues outside the β-sheet could potentially crosslink also, the major sites of crosslinking likely involve conserved aromatic residues in the RNP-1 and RNP-2 core submotifs at the center of the β-sheet, as has been shown for the RRMs of hnRNP A1 [65]. Addition of an N-terminal tag to ΔRS may exacerbate the inhibitory effect of the natural N-terminus on RNA binding, accounting for the early observations that His-tagged SF2/ASF lacking its RS domain was unable to support constitutive splicing [40], [41], [62]. Curiously, the R8E mutation, but not the V6A/I7A/R8A or R8A mutations, abrogated the inhibitory effects of the N-terminus on the ability of ΔRS to support splicing of IgM M1-M2 (Figure 1C). In a scenario in which the N-terminal extension of RRM1 prevents recruitment of ΔRS to pre-mRNA by blocking access to key RNA-binding residues on the β-sheet, substitution of the conserved arginine with an acidic residue, but not with an uncharged residue, may be sufficient to interfere with potential intramolecular interactions between the N-terminal extension preceding RRM1 and the RNA-binding surface of the protein.

We propose that in the context of the splicing reaction, the SF2/ASF RS domain normally assists in overcoming the inhibitory effect of the N-terminus, most probably by helping to recruit the RRM(s) to the RNA. Thus, one possible reason for the previously reported, apparent RS-domain-dependence of some substrates could be that in the context of these pre-mRNAs, binding of RRM1 of SF2/ASF is inhibited, due to unfavorable secondary structure of the pre-mRNA or steric block by proteins bound adjacent to the RRM1 target, or both; a hypophosphorylated RS domain might therefore assist in initial recruitment of SF2/ASF to its target, through its own charge-mediated contacts with adjacent RNA sequences, bringing in the N-terminal RRM region so that it can directly contact the RNA. In agreement with this hypothesis, using a UV crosslinking assay, we observed little difference between crosslinking of SF2/ASF and ΔNSF2/ASF to IgM M1-M2 pre-mRNA, whereas the ΔRS protein bound less efficiently to IgM M1-M2 than did either SF2/ASF or ΔNSF2/ASF; however, when the N-terminal extension was deleted from ΔRS, its binding to the pre-mRNA was greatly enhanced (Figure 3B).

### Constitutive splicing without an SR protein RS domain

Our finding that the RS domain is not required for constitutive splicing of IgM M1-M2 seemingly contradicts prevailing models about how SR proteins function to promote splicing. The previous finding that the RS domain is dispensable for splicing of some substrates but not for others [42] hinted that SR proteins may activate splicing in a manner that does not always involve SR protein RS-domain-mediated protein-protein interactions. However, because some substrates were found to require the RS domain, RS-domain-mediated recruitment functions of SF2/ASF could not be formally discounted, and were still hypothesized to occur for RS-domain-dependent substrates. Splicing in the absence of an SR protein RS domain suggests two possibilities for how the domains of SR proteins function to promote pre-mRNA splicing from the ESE position. First, the RS domain may indeed be required in some contexts for recruitment functions of SR proteins, and in contexts where the protein is active in the absence of the RS domain, the ΔRS portion of the protein may promote splicing by antagonizing the function of splicing silencers. Second, the ΔRS portion of the protein may be sufficient to recruit splicing factors to activate splicing. As the number of constitutive substrates that can be spliced without an SR protein RS domain continues to grow, it seems increasingly improbable that SR proteins lacking their RS domains can only support splicing in contexts where recruitment functions of SR proteins are dispensable. Instead, it appears more likely that protein-protein interactions occurring through the RS domain of SR proteins are not essential for recruitment of splicing factors, a finding that seems at odds with current recruitment models for SR protein function.

The traditional recruitment models for SR protein function assume that the ESE-bound RS domain of an SR protein interacts with the RS domain of another splicing component, such as U2AF^35^ or U1-70K. Much support has been garnered for the SR protein RS domain-mediated recruitment model, through experiments that employed MS2-RS domain fusion proteins as splicing activators [21], [56], [59]. However, if our ΔNΔRS protein is functioning to recruit other splicing components, it cannot be doing so through RS-RS domain interactions. Although there is evidence to suggest that SR proteins assist in the recruitment of essential splicing factors, such as U2AF^35^ or U1-70K, which themselves have RS domains, it is not clear that this recruitment requires the RS domains of both of the involved proteins. For example, the RS domain of SF2/ASF is not required for enhancement of U1 snRNP binding to alternative 5′ splice sites [66], which is presumed to occur through an interaction with the RS-domain-containing protein U1-70K. Indeed, it is unlikely that the SF2/ASF RS domain can function to recruit U1 snRNP through interactions with U1-70K, as the isolated, unphosphorylated RS domain of SF2/ASF is not sufficient for interaction with U1-70K [15]. It seems more probable that some portion of SF2/ASF other than, or in addition to, the RS domain engages in protein-protein interactions with U1-70K to recruit U1 snRNP, considering that a GST-ΔRS fusion protein can engage in RNA-independent protein-protein interactions with U1-70K [15]. Some labs have reported that at least one of the RS domains of SF2/ASF and U1-70K is required for interactions between these two proteins [13], [67], but these experiments did not demonstrate that the RS domains of both proteins were required for their interaction. The proposed recruitment function of SR proteins for which the most experimental evidence has been assembled is the model in which an ESE-bound SR protein engages via its RS domain in protein-protein interactions with the RS domain of U2AF^35^ to aid in the recruitment of U2AF^65^ to the polypyrimidine tract [3], [16]. However, several lines of evidence suggest that only one of these two U2AF RS domains is required for efficient splicing [68], [69], [70], and the RS domain of U2AF^35^ is dispensable for complementation of U2AF-depleted extract [44], leaving open the possibility that the RS domain of U2AF^35^ is likewise not needed for interaction with an ESE-bound SR protein.

It has been demonstrated that an RS domain at the position of the ESE, whether synthetic [29], [42] or authentic [56], and whether targeted there via an SR protein RRM [42], a heterologous RNA-binding domain, such as the MS2 coat protein [56], [57], or an antisense oligonucleotide [29], can function to promote splicing, whether this splicing activation occurs from the ESE through influencing the recruitment of other splicing factors or by promoting base-pairing of U snRNAs to pre-mRNA. From these experiments we may conclude that one of the primary functions of ESEs is to recruit an RS domain. However, an ESE is still required for splicing of IgM M1-M2 with our ΔNΔRS protein, which lacks an RS domain. Clearly, recruitment of an RS domain by any means to the position of the ESE can function to promote splicing, yet in conjunction with the previous report of RS-domain-independent splicing [42] our data strongly suggest that the SR protein RS domain is not required at this position to activate splicing. This apparent paradox can be resolved if we consider that the function of an SR protein may not be to recruit other splicing factors through its RS domain, but rather simply to recruit an RS domain, whether its own or the RS domain of another splicing factor. In an SR protein RS-domain-independent recruitment model, the SR protein would interact with another splicing factor, and this interaction must recruit at least one RS domain for splicing to be activated. An SR protein lacking its RS domain could activate splicing by interacting, for example, with U2AF^35^, which itself has an RS domain, or with U1-70K, which also has its own RS domain. Such a mechanism would be analogous to the situation described for U2AF^65^ and U2AF^35^, which interact with each other and both have RS domains, but only one of the two RS domains is required for splicing to occur [68], [69], [70]. Indeed, the requirements for the identity of the RS domain recruited to the ESE position to function as a splicing activator are far from stringent, as the RS domain from any SR protein [56], [59], [71], from U2AF^65^ [27], [57], [71], U2AF^35^ [57], [71], U1-70K [71], or even a synthetic RS domain [29], [57], [71] are all sufficient for this purpose.

Our data strongly suggest that SR protein RS-domain-mediated protein-protein interactions are not required for SR proteins to promote recruitment of other splicing factors. Alternatively, the ΔRS portion of SF2/ASF, which consists of two RRMs separated by a glycine-rich linker, may itself be engaging in protein-protein interactions with other proteins of the spliceosome. There are precedents for this possibility, as several other essential splicing factors have already been demonstrated to interact with each other through protein-protein interactions involving their RRMs [72], [73], [74]. Using the ΔNΔRS protein, the roles of portions of SR proteins other than their RS domains in promoting splicing can be explored in the future.

## Materials and Methods

### Cloning Procedures

The pTT3 vector [75] was employed for expression of C-terminally His-tagged SR proteins in 293-EBNA1 cells; plasmids pTT3-SF2His and pTT3-SF2ΔRSCHis code for amino acids 1-248 and 1-196 of SF2/ASF, respectively. Plasmids for expression of N-terminus mutant proteins were created by deletion or mutation of sequences in the pTT3-SF2His and pTT3-SF2ΔRSCHis plasmids, either as described in the Stratagene Quikchange Site-Directed Mutagenesis Kit manufacturer's protocol or using a site-directed mutagenesis strategy with a common reverse primer and mutagenic forward primers that overlap at their 5′ ends with the reverse primer. The protein-coding regions for all protein expression plasmids were verified by sequencing.

Plasmids containing transcription templates for IgM M1-M2 splicing substrates with mutations or deletions in the polypyrimidine tract, exonic splicing enhancer, and/or exonic splicing silencer were generated by either overlap-extension PCR or site-directed mutagenesis. Overlap-extension PCR [76] was carried out using Pfu Turbo polymerase (Stratagene), using outside primers to pSP65-µM1-M2 [58] upstream of the SP6 transcription start site and downstream of the XbaI site used for the transcription runoffs. Overlapping PCR products were cloned into the pCR-Blunt vector (Invitrogen). All transcription templates were verified by sequencing. IgMPy↑ was generated by overlap-extension PCR with pSP65-µM1-M2 as a template, using inside primers to introduce the mutant polypyrimidine tract 5′UUUUUUCCCUUUUUUUUUC3′ [21] in place of the wild-type polypyrimidine tract 5′ACACUGUCUCUGUCACCUG3′. IgMΔE was generated by overlap-extension PCR with pSP65-µM1-M2 as a template, using inside primers to delete the 23-nt enhancer 5′GAAGGACAGCAGAGACCAAGA3′ in exon M2 [23]. IgMPTB was generated by site-directed mutagenesis of the pSP65-µM1-M2 plasmid to introduce the mutant PTB site I 5′ACAUACGACAU3′ [45] in place of the wild-type site 5′UCUUACGUCUU3′. IgMPy↑ΔE was generated by overlap-extension PCR with pCR-Blunt-IgMΔE as a template, using inside primers as described above to introduce the mutant polypyrimidine tract in place of the wild-type polypyrimidine tract. IgMΔEPTB, IgMPy↑PTB, and IgMPy↑ΔEPTB were generated by site-directed mutagenesis of the pCR-Blunt-IgMΔE, pCR-Blunt-IgMPy↑ and pCR-Blunt-IgMPy↑ΔE plasmids, respectively to introduce the mutant PTB site I as described above.

### Protein Expression and Purification

SF2/ASF, ΔRS, and N-terminus mutant proteins were expressed as C-terminally His-tagged fusion proteins from the pTT3-SF2His and pTT3-SF2ΔRSCHis plasmids or derivatives of these plasmids, respectively, after transfection with polyethylenimine (PEI) into 293-EBNA1 cells [75]. 293-EBNA1 cells (Invitrogen) were maintained in suspension culture at a density of 2.5×10^5^ cells/mL in MEM Joklik's suspension modification medium with L-glutamine (US Biological) supplemented with 5% calf serum (Gibco) and penicillin/streptomycin. For transfection, 1L of cells at 2.5×10^5^ cells/mL were allowed to grow for 24 hours and then transfected by the addition of 1 mg of plasmid, 50 mL of culture medium, and 2 mg of PEI linear MW = 25,000 (Polysciences) to the suspension cell culture.

After transfection, cultures were grown for three days to allow for protein expression. Pelleted cells were washed with PBS and resuspended in a lysis buffer consisting of 1 M NaCl, 0.1% v/v Triton X-100, 20 mM β-mercaptoethanol, 50 mM TRIS-HCl pH 8.0, 10 mM imidazole, supplemented with the EDTA-free Complete Mini protease inhibitor tablet (Roche) prior to sonication. The sonicate was centrifuged at 15,000 rpm, and a 0-30% saturated ammonium sulfate cut was carried out on the supernatant, followed by another 15,000 rpm centrifugation. The second supernatant was diluted with an equal volume of lysis buffer without salt and then incubated at 4°C with Ni-NTA agarose beads (Qiagen) in batch. Beads were washed on a column with 50 volumes of lysis buffer without Triton X-100, and proteins were eluted in lysis buffer with 1 M NaCl and 300 mM imidazole and without Triton X-100. After elution from the Ni-NTA agarose column, fractions containing SR protein were combined. If the protein concentration was at least 3 mg/mL for the pooled peak fractions, proteins were directly dialyzed twice against Buffer D, consisting of 20 mM HEPES-KOH pH 8.0, 0.2 mM EDTA, 20% (v/v) glycerol, 0.4 M KCl, 1 mM DTT, and 0.5 mM PMSF. If the protein concentration was less than 3 mg/mL after combining peak fractions, proteins were denatured by dialyzing into Buffer D with 6 M urea at 0.1 M KCl, and then concentrated at 4°C to approximately 3 mg/mL using a Centricon-10 concentration device (Millipore Corporation). Concentrated proteins were refolded by sequential dialyses in Buffer D containing 3 M urea and 0.4 M KCl, 1.5 M urea and 0.4 M KCl, 0.75 M urea and 0.4 M KCl, and finally into Buffer D.

### In Vitro Splicing Assays

Splicing substrates were transcribed from plasmid templates linearized with XbaI using SP6 RNA polymerase (Promega), essentially as described in [77], except that G(5′)ppp(5′)G cap analog (NEB) was used instead of ^7m^GpppG cap analog. All transcripts were gel-purified. HeLa cell cytoplasmic (S100) extracts were prepared as described in [78]. *In vitro* splicing assays were carried out essentially as described in [79]. Briefly, 10-µL reactions containing 0.5 mM ATP, 20 mM creatine phosphate, 20 mM HEPES-KOH pH 7.3, 2.6 % (w/v) polyvinyl alcohol, 1.6 mM MgCl_2_, 20 fmol α^32^P-UTP labeled splicing substrate, 30% (v/v) S100, and 16 pmol SR protein were set up on ice. For S100 complementation reactions with SR proteins in 0.4 M KCl Buffer D, the final salt concentration for the splicing reaction was adjusted to 60 mM using Buffer D without salt. Splicing reactions were incubated at 30°C, followed by phenol extraction and ethanol precipitation. RNA was resuspended in formamide/bromophenol blue/xylene cyanol FF loading dye, and separated in a 5.5% acrylamide/8.3 M urea gel. Bands were visualized by autoradiography using X-OMAT film (Kodak) or by exposure to a FUJI PhosphorImager screen and analysis with an Image Reader FLA-5100 (FujiFilm Medical Systems, Stamford, Connecticut, United States). Percent splicing was calculated as [mRNA/(mRNA+pre-mRNA)]×100.

### UV Crosslinking

Protein binding was performed in 20-µL reactions containing 32 pmol of SR protein or BSA, 80 fmol of uncapped IgM M1-M2 RNA labeled with all four NTPs, in reaction buffer conditions with final concentrations of 1.6 mM MgCl_2_, 20 mM HEPES-KOH pH 7.3, 0.5 mM ATP, 20 mM creatine phosphate, and 60 mM KCl. RNA was denatured prior to incubation with protein; RNA mixes containing 80 fmol of labeled IgM M1-M2, MgCl_2_, and water were assembled at 4°C, heated at 95°C for five minutes, and then returned to ice. 32 pmol of BSA or SR protein in Buffer D was then added to each reaction along with the other splicing buffer components, and reactions were incubated at 30°C for 30 minutes. Binding reactions were spotted onto parafilm and placed on ice prior to UV crosslinking at 0.864 J/cm^2^ in a Spectrolinker XL-1000 UV Crosslinker (Spectronics Corporation). Reactions were returned to Eppendorf tubes, and 3 µL of 27 mg/mL RNAse A and 2 µL of 1000 U/µL RNAse T1 (Roche) were added prior to incubation at 37°C for 15 minutes. Proteins and RNA were separated by 12% SDS-PAGE prior to visualization by autoradiography and phosphorimaging.
